# Providing a Prostate Cancer Detection and Prevention Method With Developed Deep Learning Approach

**DOI:** 10.1155/proc/2019841

**Published:** 2025-05-08

**Authors:** Alireza Zarei, Elias Mazrooei Rad, Shahryar Salmani Bajestani, Seyyed Ali Zendehbad

**Affiliations:** ^1^Department of Engineering, Faculty of Biomedical Engineering, Apadana Institute of Higher Education, Shiraz, Iran; ^2^Biomedical Engineering Department, Khavaran Institute of Higher Education, Mashhad, Iran; ^3^Department of Biomedical Engineering, Mashhad Branch, Islamic Azad University, Mashhad, Iran

**Keywords:** deep learning, manifold learning, medical image processing, prostate cancer detection, prostate cancer prevention

## Abstract

**Introduction:** Prostate cancer is the second most common cancer among men worldwide. This cancer has become extremely noticeable due to the increase of prostate cancer in Iranian men in recent years due to the lack of marriage and sexual intercourse, as well as the abuse of hormones in sports without any standards.

**Methods:** The histopathology images from a treatment center to diagnose prostate cancer are used with the help of deep learning methods, considering the two characteristics of Tile and Grad-CAM. The approach of this research is to present a prostate cancer diagnosis model to achieve proper performance from histopathology images with the help of a developed deep learning method based on the manifold model.

**Results:** Similarly, in addition to the diagnosis of prostate cancer, a study on the methods of preventing this disease was investigated in literature reviews, and finally, after simulation, prostate cancer presentation factors were determined.

**Conclusions:** The simulation results indicated that the proposed method has a performance advantage over the other state-of-the-art methods, and the accuracy of this method is up to 97.41%.

## 1. Introduction

The most common type of cancer among men is prostate cancer (PCa) [1]. PCa is the third most common cancer among Iranian men after stomach cancer and skin cancer. According to these statistics, 16.93% of men in Iran get this cancer [2]. Some people are faced with the question of how to prevent PCa. Race and genetic factors play an important role in the occurrence of this disease [3]. Keeping the body healthy following the principles of health with increasing age and treating current problems and diseases will reduce the risk of PCa. PCa is a growing tumor that usually does not put pressure on any part and will not cause pain. For this reason, this disease may not have any early warning signs for a long time. However, advanced PCa often causes symptoms. Some symptoms of PCa can be caused by other diseases, so the person will need to be examined. Among the symptoms of PCa in men, we can mention urinary problems, sexual problems, pain, and numbness [4]. Following some tips will reduce the risk of PCa. The main cause of the appearance of this cancer is multiple factors such as age, inheritance, job, nutrition, and the use of various drugs that are effective in causing this disease. The presence of PCa in the family causes other people to be at risk of developing this type of cancer. Also, as the age increases, the risk of developing this type of cancer increases. Therefore, these people can prevent this cancer by following a proper diet, regular sports activities, and periodic checkups to reduce the risk of contracting it. The prevention of PCa strongly depends on several lifestyle elements which include what people eat and their exercise routine and how their hormones function. Scientific research indicates that PCa progression risks decrease when people eat foods containing antioxidants like tomato lycopene and fish-derived omega-3 fatty acids [5]. The incidence of PCa decreases when people stay active physically and maintain healthy weights because insulin-like growth factors remain in check [6]. Some studies connect hormonal abuse in sports to PCa risk elevation from altered testosterone levels, but additional extensive clinical trials must confirm this connection [7]. Effective activities in preventing PCa include maintaining proper weight, avoiding smoking, regular exercise, maintaining sexual activity, and regular checkups and tests [1–4]. A suitable diet for PCa prevention includes the use of cabbage, coffee, tomatoes, and other red fruits, vitamin D, and the consumption of soybeans [1–4]. Digital rectal examination and prostate-specific antigen (PSA) tests are used to accurately diagnose PCa. Since PSA is not organ- and disease-specific, multiparametric magnetic resonance imaging (mpMRI) is used to reduce unnecessary biopsies. Artificial intelligence has been widely used as one of the solutions to diagnose and prevent PCa in recent years. Deep learning methods and artificial neural networks have better results than other machine learning methods in the evolution of the methods provided for this purpose. There are some powerful studies in the field of PCa diagnosis with the help of deep learning methods from histopathology images [8], MRI images [9], a combination of MRI and CT scan images [10], MRI images and pathology [11], and radiation therapy [12]. It has been studied abundantly, and each of them has presented studies and methods based on different deep learning models. This research also tries to provide a method to diagnose and prevent PCa with the help of a developed deep neural network method. For this purpose, it is important to review some previous methods and observe their results. Various studies have been presented in the field of PCa diagnosis and prevention from image data with a deep learning approach. In [13], the author proposed an image segmentation method for radiation therapy planning to diagnose and prevent PCa from CT scan images with the help of U-NET deep learning with an accuracy of 93.78%. Likewise, in [14], a deep learning method based on two S-Mask R-CNN and Inception-v3 structures was presented for detecting PCa from ultrasound images with an accuracy of 95.14%. In [15], a hybrid model was presented and extracted for accurate mpMRI test interpretation and PI-RADS rating prediction with the help of three deep neural network architectures, including MobilenetV2, EfficientNetB0, and Darknet53 [16]. The accuracy of this method is 96.09%. In [17], deep learning–based reconstruction (dDLR) has been used for imaging feasibility in seventeen PCa patients who underwent DWIBS and MRI imaging, and its accuracy is 96.16%. In [18], the system using the YOLO object detection algorithm from 1776 labeled biopsy images was used for the automatic detection of PCa, which has a detection accuracy of about 93.774%. In [19], the prediction of the therapeutic dose to prevent PCa from 160 patients has been performed with the U-NET deep neural network, and the accuracy of this method is up to 96.46%. In [20], 85 data patients have been used to develop a deep learning algorithm for histopathology diagnosis and Gleason grading of PCa biopsies, which is predicted to be 94% accurate. Other studies for detecting PCa from image data proposed with the help of other deep learning methods include recurrent deep learning (RNN) [21], convolutional neural network (CNN) [22, 23], PSA-Net neural network [20], and multichannel convolutional neural network (MCCNN) [24]. Existing diagnostic methods encounter multiple challenges including poor accuracy rates in PCa diagnosis, insufficient examination of preventive strategies before and after diagnosis, and the absence of standardized datasets for detection and high execution times from previous approaches. The development of deep learning models for superior performance is paired with minimal exploration of manifold learning integration for deep learning applications. High-dimensional histopathology data become easier to interpret through manifold learning because it captures intrinsic data relationships which could lead to improved accuracy and generalization. The reviewed research [13–24] has several main weaknesses: (1) low accuracy in PCa diagnosis, (2) lack of examination of problems in prevention methods before and after PCa diagnosis, (3) lack of suitable standard data for detection, and (4) computational complexity and high execution time in previous methods. Therefore, covering and removing these weaknesses requires new methods, and in this research, we propose a new method to cover the weaknesses of the previous methods based on developed deep learning methods.

## 2. Method and Materials

**Figure 1:**
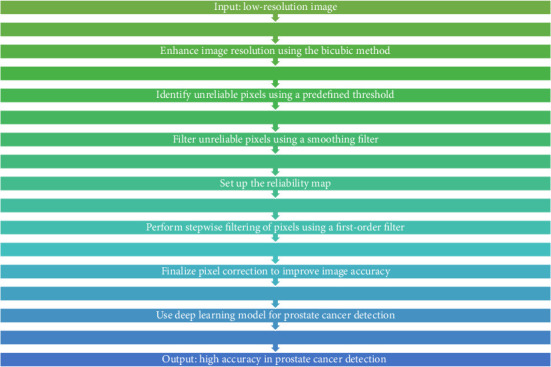
Overall framework of the proposed method.

The deep neural network of this research is a self-organizing method that first enhances the low-resolution depth map as a prototype and then indicates the unreliable pixels in the high-depth map that are mostly around the image boundary. Determining unreliable pixels is relatively simple. A pixel is considered unreliable if the difference between the maximum and minimum values in a reference window is greater than a predetermined threshold for each pixel of the initially interpolated image. Unreliable pixels are then filtered out with a convolution iteration, where unreliable pixels are processed around reliable pixels in each iteration, and then, the filtered pixels are considered valid samples. Similarly, the innovation of the proposed approach includes the use of reliability mapping to correct the unreliable pixels and the threshold adjustment by determining the order. According to Figure 1, the overall framework of the proposed method is clear. The proposed method benefits from Figure 1 which demonstrates the step-by-step process showing how reliability mapping together with filtering techniques enhances accuracy levels. It is clear that there is still much space for improvement despite the good performance of the low-complexity manifold setup. First, the generation of reliability maps is not carefully considered. The threshold adjustment filters only the unreliable pixels, while the reliable pixels remain unchanged. Therefore, it is very important to find unreliable pixels in preprocessing. Second and most importantly, the order of the filter is very important to the final results. However, manifold regularization easily determines the phase with a set of approximately reliable pixels in a step-by-step manner, which results in the order of the filter being completely dependent on reliable mapping. Instead, it is necessary to specify the filtering command carefully. The proposed scheme solves these two problems by adding a deep neural network–based training structure, which will lead to a significant performance improvement.

Most of the image measurement methods prefer to use the two-part scheme to obtain an initial upsampled image due to its sufficient performance and simplicity. The manifold setup also uses the bicubic method for initialization and reliability mapping, and all steps are performed with a bicubic upsampled image. However, it can be seen that the cubic spline method (which is similar to the manifold adjustment) often causes over or underprocessing around the edges of the image, and this can be a significant issue when filtering the image. The proposed filter is applied in a stepwise manner, where reliable neighboring pixels play an important role in determining unreliable pixels. Therefore, it is important to keep reliable pixels as close to the boundaries as possible. In the proposed scheme, we will simply replace them with two-way upsampled data. It has been proven that the bilinear method is simpler but gives a weaker result. Nevertheless, the bilinear method does not cause any excessive problems or solve the problem, and for the proposed design, it is more suitable to adopt the bilinear method for the guidance filter in the manifold adjustment. The proposed scheme only applies to unreliable pixels. Therefore, it is very important to correctly classify the reliable and unreliable pixels in the initial sparse depth mapping. The main obstacle in deep learning–based filtering involves understanding how manifold adjustment and reliability mapping together influence the final output. The authors added visual depictions to enhance the interpretability of each processing stage. In this case, the pixel is classified as a reliable pixel instead. To solve this problem, this research proposes a simple first-order filter method because it can deal with the directional patterns of image contents. In other words, a first-order derivative edge filter can distinguish fine details with zero crossings, which remain reliable segments. This method is especially effective when the scale factor is large because the fine details are very unpredictable. A major contribution of early filtering is the use of manifold tuning, i.e., instead of filtering unreliable pixels all at once, filtering is performed sequentially. This means that the newly filtered pixels in the previous steps participate in the filtering process of the next step. For the 'th iteration, all the unreliable pixels near the reliable pixels are searched, and the order of these pixels set. Then, these pixels are marked as reliable for future iterations. After a sufficient number of iterations, all pixel orders are determined to be unreliable. This step is similar to geodesic distance conversion, where the unreliable pixels are filtered based on their neighboring reliable pixels in each step, and the newly updated pixel values are distributed in the center of the unreliable regions. Therefore, the midline or curve between the two boundaries in the initial confidence map is the boundary of the image. However, it is possible that the true edges are not the untrusted parts. When classifying, some pixels are only estimated by filtering information from the other side. The proposed approach in the manifold adjustment section, considering the color similarity and second-order slope of the depth mapping, uses a guided filter, which reduces the above problem, but this is not a fundamental solution. The proposed method in the manifold adjustment section suggests a solution that uses confidence mapping. The proposed approach combines multiple precise steps which allow accuracy enhancement while maintaining efficiency in calculation. The diagram in Figure 1 shows the relationship between manifold tuning and deep learning–based reliability filtering to explain their integration. Instead of determining the order of the filter based on the geometric proximity, the similarities of intensity mapping, depth mapping, and edge directions are considered. In this approach, confidence mapping in vector form is proposed to determine the rank. In mathematical language, the confidence value in the position of the pixel *x* = (*x*_1_, *x*_2_) is in the form of equation (1).

(1) $$\begin{array}{c} \overset{¯}{C}\left(x\right)=\left[\begin{array}{c} C\left(x;+\nabla T\left(x\right)\right)\\ C\left(x;-\nabla T\left(x\right)\right) \end{array}\right]. \end{array}$$

In this regard, *T* represents the distance map and ∇ is the gradient operator. Therefore, ∇*T* represents the propagation direction for the filter. Likewise, the confidence map includes confidence values for both directions of propagation. Confidence values for valid pixels are set as [1, 0]^*T*^. The confidence values for unreliable pixels with two propagation directions are obtained independently by equation (2):

(2) $$\begin{array}{c} C\left(x;\nabla x\right)=\frac{1}{K_{C}}\underset{y\in W\left(x\right)}{\sum }a^{n}B\left(x,y\right)G\left(x,y;\nabla_{x},\nabla_{y}\right)C\left(y;\nabla_{y}\right), \end{array}$$

where *W*(*x*) returns the reliable neighbor pixels and *K*_*C*_ is the normalization factor. ∇_*x*_ and ∇_*y*_ represent ±∇*T*(*x*) and ±∇*T*(*y*), respectively. *B* and *G* are defined as equations (3) and (4):

(3) $$\begin{array}{c} B\left(x,y\right)=\mathrm{Exp}\left(-\gamma_{I}{\left\|I\left(x\right)-I\left(y\right)\right\|}^{2}\right)\mathrm{Exp}\left(-\gamma_{D}{\left\|D\left(x\right)-D\left(y\right)\right\|}^{2}\right), \end{array}$$

(4) $$\begin{array}{c} G\left(x,y;\nabla_{x},\nabla_{y}\right)=\max\left(\frac{\nabla_{x}^{T}\nabla_{y}}{\left|\nabla_{x}\right|\left|\nabla_{y}\right|},\delta \right), \end{array}$$

where *δ* ≥ 0 and *I* and *D* return mapping values and mapping depth, respectively. Here, *B* measures the consistency of the depth map and its corresponding color intensity, where *γ*_*D*_ and *γ*_*I*_ set the parameters. This means that confidence values are degraded when there is a large change in depth mapping or color values during propagation. *G* measures the consistency of the boundary normal vector that controls the confidence propagation, which only works if both positions have similar directions, i.e., if two normal vectors have opposite or orthogonal directions, the confidence value is tripled. Next, *a* is the decaying factor, which is 0 ≤ *a* < 1, and the confidence value decreases by *a* when the number of publications increases with sign *n*. It can be assumed that the proposed method in the manifold setting section generalizes the training model in deep learning, that is, by setting *γγ*_*I*_ = *γ*_*D*_ = 0 and *δ* = 1, and it becomes the same as the previous manifold in the mentioned articles. In the proposed plan to adjust the manifold, *a* = 0.8, *γ*_*I*_ = 0.1^−2^, *γ*_*D*_ = 0.01^−2^, and *δ* = 0 are determined. Next, the deep neural network starts working. The deep neural network is used to extract the feature map, which is aggregated by a global summation layer, into a compact view of fixed length. Finally, this profile is first projected with a fully connected layer, and then, the *B* and *G* parameters are normalized to compare the images with the raster output. All the components of a deep neural network, including the aggregation layer, are different, which makes it trainable to the end. For the deep neural network part, an image color histogram captures the color distribution in an image and does not include correlation space information. In the beginning, there is an image called *I*, which is processed in the form of rows and columns and is known as *n* × *n*. The colors of the input image *I* are quantized into *m* colors and displayed as *c*_1_,…, *c*_*m*_. *m* is a constant value obtained from the result of the analysis at runtime. A pixel is displayed as *p* = (*x*, *y*) ∈ *I*, where *x* and *y* are the rows and columns of the image and are part of the input image, and *I*(*p*) refers to the color in the image. There is also the relationship *I*_*C*_ = {*p*|*I*(*p*) = *c*} and it is considered as an assumption. Therefore, *p* ∈ *I*_*c*_ is similar to *p* ∈ *I*, *I*(*p*) = *c*. In order to simplify the work, *L*_*∞*_ norm has been used to measure the distance between image pixels. For example, for the pixel *p*_1_ = (*x*_1_, *y*_1_), *p*_2_ = (*x*_2_, *y*_2_), the relationship is determined as equation (5):

(5) $$\begin{array}{c} \left|p_{1}-p_{2}\right|=\max\left\{\left|x_{1}-x_{2}\right|,\left|y_{1}-y_{2}\right|\right\}. \end{array}$$

The value of *h* or the histogram of the input image or *I* for *i* ∈ |*m*| is determined that it is written and proved as equation (6).

(6) $$\begin{array}{c} {h_{c}}_{i}\left(I\right)=n^{2}.Pr_{p\in I}\left[p\in {I_{c}}_{i}\right]. \end{array}$$

For each pixel in the image, *h*_*c*__*i*_(*I*)/*n*^2^ provides a probability value that the color of the pixel is *c*_*i*_. The distance criterion is prioritized and expressed as *d* ∈ [*n*]. Then, the correlation of the input image or *I* is determined as *i*, *j* ∈ *m*, *k* ∈ [*d*] and expressed as equation (7):

(7) $$\begin{array}{c} \gamma_{c_{i},c_{j}}^{\left(k\right)}\left(I\right)=Pr_{p_{1}\in {I_{c}}_{i},p_{2}\in I}\;\left[\left.P_{2}\in {I_{c}}_{i}\;\right|\left|\;p_{1}-p_{2}\right|=k\right]. \end{array}$$

Each pixel *c*_*i*_ in the image provides a series of possibilities in the form of *γ*_*c*_*i*_⁣,⁣*c*_*j*__^(*k*)^ where each pixel is at distance *k*, outside the pixel in the image. It should be noted that the correlation size is *O*(*m*^2^*d*) as an assumption. Image autocorrelation *I* deals with maintaining only the spatial correlation between detected colors and is determined as

(8) $$\begin{array}{c} a_{c}^{\left(k\right)}\left(I\right)=\gamma_{c_{i},c_{j}}^{\left(k\right)}\left(I\right). \end{array}$$

This image information is a subset of correlation and requires *O*(*md*) space. When *d* is chosen as the correlation, there is a need to address the color table to perform depth recovery during training in the aggregation layer. The result of a large *d* value will be computationally intensive and require large storage space in the color table. A small *d* value may reduce the quality of the features. Data normalization is included in the parameter initialization section. Normalization makes the Euclidean distance between [0, 1]. After this step, the similarity threshold should be calculated. This value is used in the memory cell identification step. Equation (9) is used to calculate the similarity, which is the average similarity (Euclidean distance) between all classes of depth mapping in the image:

(9) $$\begin{array}{c} I=\frac{\sum_{i=1}^{n}\sum_{j=i+1}^{n}\text{affinity}\left(Ag_{i},Ag_{j}\right)}{n\left(n-1\right)/2}. \end{array}$$

In the next part, which is the determination of memory cells and the generation of cells for the purpose of celling the image and points, an area growth method is also applied to these cells, a candidate memory cell to be placed or replaced in the cell set. For this purpose, it is necessary to train the memory cell that has the most stimulation to the distorted or noisy parts. This research considers this situation which leads to small *d*. In the following, the nonlocal modes of manifold with a desired principal function will be used. The nonlocal manifold model *M*_*nl*_ is designed to exploit the nonlocal similarity relationship of pixels. For each pixel *i* in *f*, an *s* × *s* patch of *R*_*i*_*f* is extracted around it, where *R*_*i*_ is a matrix of extraction of a patch from *i*. Accordingly, the fitting function is in the form of equation (10):

(10) $$\begin{array}{c} \phi_{f}\left(i\right)=R_{i}f\in M_{nl}\subset \iota^{2}\left(\Omega_{s\times s}\right)\subset R^{s^{2}}, \end{array}$$

The set of patches {*R*_*i*_*f*⁣}_*i*∈Ω_ can be thought of as a set in a high-dimensional space whose geometry shows the nonlocal properties of *f*. It should be noted that this research only uses depth information in the definition of the nonlocal multilocal model. For the nonlocal curvature *M*_*nl*_, the features are the same as the local patches, and accordingly, the diffusion kernel is defined as equation (11):

(11) $$\begin{array}{c} W_{nl}\left(i,j\right)=\exp\left(-\frac{{\left\|R_{i}f-R_{i}f\right\|}_{2}^{2}}{2\sigma^{2}}\right). \end{array}$$

In this regard, ‖*R*_*i*_*f* − *R*_*i*_*f*‖_2_^2^ measures the similarity between two patches *R*_*i*_*f* and *R*_*i*_*f* around *i* and *i*. Similar to the local manifold, the Laplace matrix can also be defined for the nonlocal manifold, which is in the form of equation (12):

(12) $$\begin{array}{c} L_{nl}=D_{nl}-W_{nl}. \end{array}$$

The nonlocal features of patch-based manifolds can be used to construct a highly data-adaptive orthogonal basis for extracting imaged patterns. In particular, since *L*_*nl*_ is symmetric and real, it can be decomposed into orthogonal vectors with real eigenvalues, which are in the form of equation (13):

(13) $$\begin{array}{c} L_{nl}=U\wedge U^{T}, \end{array}$$

where *U* = {*u*_*k*_} is a set of orthogonal eigenvectors. The negative eigenvalues {*η*_*k*_} are related to the entries of the diagonal matrix ∧. The eigenmatrix *U* forms the spectral basis of the manifold, which is consistent with the signal in the manifold. Negative eigenvalues {*η*_*k*_} can be interpreted as graph frequencies with small *η*_*k*_ corresponding to low frequencies and eigenvectors *U* as frequency components of the corresponding graph.

## 3. Results and Discussion

**Figure 2:**
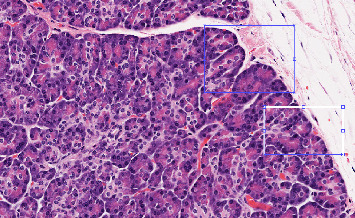
Input sample and some defective parts.

**Figure 3:**
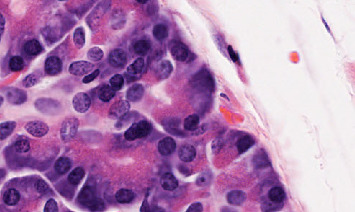
Magnified view of the defected region.

**Figure 4:**
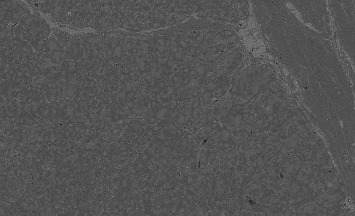
Contrast adjustment in manifold representation.

**Figure 5:**
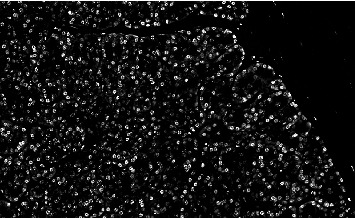
Detection of tile features.

**Figure 6:**
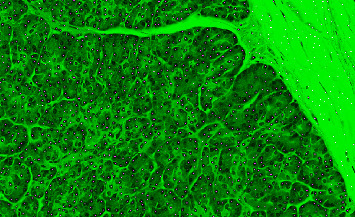
Detection of Grad-CAM features.

The simulation was performed in a MATLAB environment from 1000 histopathology image samples from the clinical dataset in Iran from 2016 to 2022. The dataset contains tissue samples that represent both normal benign regions and cancerous malignant regions of prostate tissue. The clinical population representation in the dataset exists, but the data can contain biases because of regional imaging limitations and insufficient patient demographic diversity. The dataset employed in this research does not function as a standard benchmark for PCa image examination. The restricted dataset creates obstacles to perform direct comparisons of the proposed method with alternative methods. The analysis should note possible sources of bias which include unbalanced classes together with inconsistent imaging conditions. To enhance model generalization, the model needs strategies for data augmentation and balanced sampling to address existing biases. Additional performance metrics including precision, recall, *F*1-score, and AUC will be used to conduct a complete evaluation of the model in order to achieve a comprehensive assessment of its performance. Evaluation of possible overfitting issues will be addressed in future research. All images will be input in developed deep learning based on manifold methods, 70% are training, and 30% are testing. Our obtained results in the simulation are shown in Figures 2, 3, 4, 5, and 6. The proposed model demonstrates promising results, but its performance can be made more stable by including k-fold cross-validation methods or utilizing publicly available datasets as an additional test. Future research will investigate this aspect to improve the general applicability of the proposed system.

**Figure 7:**
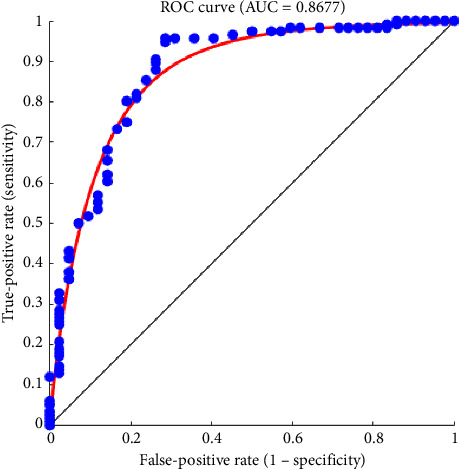
ROC curve and AUC for model performance evaluation.

The true-positive and false-positive rates tradeoff can be better understood through the ROC curve. The AUC value is 0.8677 as shown in Figure 7 which indicates that there is a minor error as the AUC is below 1. This type of mismatch can arise when certain cancer masses resemble noncancerous regions. These errors lead to scoring adversely; however, this AUC still demonstrates strong classification performance. It is robust, and the AUC being so close to 1 is a key indicator of a model that performs well.

We can see that the considered features, Tile and Grad-CAM, were extracted from the eight selected regions, and then, they show benign–benign tiles randomly selected in regions and cancer–benign tiles randomly selected from the region, respectively. The corresponding Grad-CAM plots highlight important structures related to cancer diagnosis.

The evaluation requires additional investigation to fully understand how Grad-CAM and Tiles features contribute to the classification process. The visual explanations generated by Grad-CAM indicate important image regions while the Tiles method helps organize and segment histopathological patterns to make the model more interpretable. The complementary nature of Grad-CAM with Tiles features in malignancy detection needs deeper evaluation to enhance knowledge about the stability of the proposed approach. Researchers need to analyze how the proposed features affect classification performance in systematic studies that can use ablation studies and feature importance analysis. In the end, we can compare our method in terms of accuracy evaluation criteria with recent methods, but it should be noted that we can compare methods in the same condition, for example, the same parameters and the same dataset. So, due to the different datasets used in recent methods and this study, because there is no standard PCa image dataset, our comparison is also about the parametric differences in deep learning methods, which are represented in Table 1. The provided table presents accuracy values from recent research studies for comparison purposes. The diverse methods and evaluation standards used in previous studies prevent direct quantitative analysis between them. A better evaluation requires the consideration of computational complexity together with execution time as well as performance metrics that include precision, recall, and AUC. Future research should create a standardized PCa image dataset to allow accurate comparison between studies because no consistent dataset currently exists.

Simulation results carried out on a dataset of 1000 histopathology images show the possibility of the proposed deep learning model based on manifold methods to detect significant features for PCa diagnosis. We can see different key stages of the analysis in Figures 2, 3, 4, 5, and 6: identifying defective regions (Figures 2 and 3), improving contrast in manifold space (Figure 4), and finding relevant image features using Tiles and Grad-CAM methods (Figures 5 and 6). In particular, the Grad-CAM plots highlight important cancer-related structures for more accurate classification of benign and malignant regions. Finally, the model reached 97.41% accuracy, outperforming recent methods such as Nemoto et al. [13] and Liu et al. [14], whose accuracies were between 93.74% and 96.46%. This suggests that the combination of manifold techniques and deep learning greatly increases the model's ability to identify cancerous features in histopathology images. Nevertheless, while comparing results, variations of datasets and parameters in disparate studies must be taken into consideration since there is no standard dataset for PCa imaging. However, this adds a level of complexity to the direct comparisons, but the robustness of the proposed approach under these experimental conditions is highlighted.

Manifold learning has considerable benefits compared to conventional deep learning approaches in detecting PCa as it improves feature extraction, manages nonlinear relationships, and performs considerably well with smaller datasets. Its combinability with deep learning also allows for boosting accuracy and generalization of the model at the same time improving the interpretability of the system. By utilizing the benefits of deep learning and manifold learning, the latter can serve as an enhancement for other models meant for PCa detection.

## 4. Conclusion

The prostate is a small gland that is located under the male bladder and is part of the reproductive system. Some men develop PCa at an advanced age. What is the cause of PCa, and what factors increase the risk of this cancer? This is a topic that we discussed in this article. In the following, we will point out the factors that have a greater impact on getting this disease; also, we propose an intelligent method for automatic PCa detection. There are many techniques in PCa detection from clinical image datasets, and deep learning techniques and methods have gained better results in recent years. Our results indicated Tiles and Grad-CAM extraction, which has an accuracy of about 97.41%, which was better in comparison to recent deep learning techniques and methods for PCa detection. In the end, we should note that based on our obtained results, two main factors of PCa prevention are sexual activities and not using sports supplements.

## Figures and Tables

**Table 1:** Comparative analysis of deep learning model accuracies.

Reference	Accuracy (%)	Computational complexity
Nemoto et al. [13]	93.78	Moderate due to large dataset and U-Net model complexity; requires significant memory and processing power for training and segmentation
Liu et al. [14]	95.14	High due to the use of two deep learning models (S-Mask R-CNN and Inception-v3), which require substantial computational resources for both segmentation and classification tasks, including forward and backpropagation processes
Liu et al. [15]	96.01	High due to the use of a 10-CNN ensemble model, which involves substantial computation for training and optimization over a large dataset
Yildirim et al. [16]	96.09	High due to the use of three deep learning architectures (MobilenetV2, Efficientnetb0, Darknet53) for feature extraction, followed by neighborhood component analysis (NCA) to reduce feature redundancy
Tajima et al. [17]	96.16	Moderate due to the use of deep learning–based reconstruction (dDLR) for image denoising. The method involves significant computation for image processing and denoising, but the dataset size is relatively small, so the complexity is manageable for this specific task
Salman et al. [18]	93.74	High due to the use of the YOLO algorithm for both region detection and classification, which involves substantial computation for training the model on a large dataset, including data augmentation
Lempart et al. [19]	96.46	High due to the use of a modified U-Net trained on triplets, which requires considerable computational resources for training on a large dataset
Kott et al. [20]	94	High due to the use of a deep residual convolutional neural network (CNN), requiring significant computational resources for training on a large number of image patches (14,803 patches) and performing multilevel classification
Our method (developed deep learning based on manifold)	97.41	High due to the use of deep learning methods, particularly the manifold model, which requires significant computational resources for training on histopathology images

## Data Availability

The data that support the findings of this study are available from the corresponding author upon reasonable request.
